# Comparative RNA-Seq Analysis between Monoecious and Androecious Plants Reveals Regulatory Mechanisms Controlling Female Flowering in *Cucurbita pepo*

**DOI:** 10.3390/ijms242417195

**Published:** 2023-12-06

**Authors:** María Segura, Alicia García, Álvaro Benítez, Cecilia Martínez, Manuel Jamilena

**Affiliations:** Department of Biology and Geology, Agri-Food Campus of International Excellence (CeiA3) and Research Center CIAIMBITAL, University of Almería, 04120 Almería, Spain; msm423@ual.es (M.S.); alicia.garcia@ual.es (A.G.); alvarobm@ual.es (Á.B.)

**Keywords:** female flowering, ethylene, hormones, transcription factors

## Abstract

In the monoecious *Cucurbita pepo*, the transition to female flowering is the time at which the plant starts the production of female flowers after an initial male phase of development. Ethylene plays an essential role in this process since some ethylene deficient and ethylene-insensitive mutants are androecious and only produce male flowers. To gain insight into the molecular mechanisms regulating the specification and early development of female flowers, we have compared the transcriptomic changes occurring in the shoot apices of WT and androecious ethylene-insensitive *etr1b* mutant plants upon female flowering transition. There were 1160 female flowering-specific DEGs identified in WT plants upon female flowering, and 284 of them were found to be modulated by the ethylene-insensitive *etr1b* mutation. The function of these DEGs indicated that female flower specification depends on the adoption of a transcriptional program that includes previously identified sex-determining genes in the ethylene pathway, but also genes controlling the biosynthesis and signaling pathways of other phytohormones, and those encoding for many different transcription factors. The transcriptomic changes suggested that gibberellins play a negative role in female flowering, while ethylene, auxins, ABA and cytokinins are positive regulators. Transcription factors from 34 families, including *NAC*, *ERF*, *bHLH*, *bZIP*, *MYB* and *C2H2/CH3*, were found to be regulating female flowering in an ethylene-dependent or -independent manner. Our data open a new perspective of the molecular mechanisms that control the specification and development of female flowers in *C. pepo*.

## 1. Introduction

*Cucurbita pepo*, commonly known as squash or pumpkin, is one of the most cultivated and economically important crops worldwide. It is a monoecious species characterized by the production of single female or male flowers at each plant node. Two different phases of sexual development can be distinguished in squash adult plants: (i) an initial male phase where the plant produce only male flower, and (ii) a mixed phase characterized by the alternating production of female and male flowers. Some varieties, also show a third female phase in which the plant produces predominantly or exclusively female flowers [1]. The transition between the first and the second phase of development is called the female flowering transition. Once female flowering starts, sex-determination mechanisms regulate whether the floral meristem is determined into a male or a female flower. In plants, unisexual female and male flowers are known to be determined by mechanisms that trigger the abortion of stamen or carpels from a floral meristem that initiates it development as bisexual [2].

Plant sex determination mechanisms are well known to be regulated by environmental and hormonal factors [3,4]. In cucurbit species, the control of female flowering transition and sex determination in each single floral meristem is known to be controlled by the phytohormone ethylene [5,6,7,8]. After different functional studies based on the isolation and characterization of different mutations in ethylene biosynthesis and signaling genes, the role of this phytohormone in the control of sex determination has been elucidated in cucurbits like cucumber and melon, but the initiation of female flowering transition by ethylene is still unclear in *C. pepo*. The ethylene biosynthesis genes *CmACS11* in melon [9] and *CsACO2* in cucumber [10] are required for carpel development in female flowers. The activity of these two genes represses the transcription factor WIP1, which is crucial for carpel abortion in cucumber, melon and watermelon, enabling the development of male flowers [11,12,13]. Loss of function mutations (LOF) in *ACS11* and *ACO2* convert female into male flowers and monoecy into androecy, and LOF of *WIP1* convert male flowers into female flowers and monoecy into gynoecy. Recently, it has been described that WIP1 promotes the development of male flowers by repressing the transcription of the carpel identity gene *CRABS CLAW (CRC)* through a TOPLESS (TLP)-mediated histone deacetylation mechanism, leading to androecious plants [14]. The female flower development also requires the activation of the ethylene biosynthesis orthologous genes *CsACS2* in cucumber, *CmACS7* in melon, *CitACS4* in watermelon, and *CpACS27A* and *CpACO1* in zucchini, which have a pivotal role in the arrest of stamen development. Loss of function mutations in these genes convert female into bisexual or hermaphrodite flowers and monoecy into andromonoecy [7,15,16,17,18]. The termination of stamen abortion has recently been attributed to the transcription factor *CmHB40*, which is influenced by the ethylene produced by *CmACS7* [19]. Ethylene perceived by the ethylene receptors is also crucial for female flowering development. By exploiting an EMS mutant collection in *C. pepo*, four ethylene receptor mutants were identified in the genes *CpETR1A* (*etr1a* and *etr1a-1*), *CpETR1B* (*etr1b*) and *CpETR2B* (*etr2b*), that lead to andromonoecy or androecy depending on the ethylene insensitivity conferred by each mutation [20,21].

Although the role of ethylene in sex expression is well documented [1,22,23], the role of other hormones in *C. pepo* sex expression remains unclear. The described feminizing effect of auxin and brassinosteroids in cucurbits appears to be mediated by ethylene [24,25]. In contrast, the exogenous application of gibberellin has been shown to produce a higher ratio of male flowers [26], but the molecular mechanisms in *C. pepo* are not well understood. Additionally, it has been reported an alternative regulation pathway independent of ethylene in cucumber [27].

To gain insight into the molecular mechanisms controlling sex determination in *C. pepo*, in this paper we have exploited the ethylene-insensitive mutation *etr1b*, that suppresses female flowering transition, converting the normal monoecy of squash into androecy [20]. By comparing the transcriptomic changes occurring in the apical shoots of WT and *etr1b* plants during the male and female phases of development, we identified a number of genes that are not only involved in ethylene biosynthesis and signal transduction pathways, but also genes encoding for transcription factors and in the biosynthesis and signaling pathways of phytohormones other than ethylene.

## 2. Results

### 2.1. Transcriptome Sequencing of WT and Etr1b Apical Shoots at M0 and M1 Stages of Development

**Figure 1:**
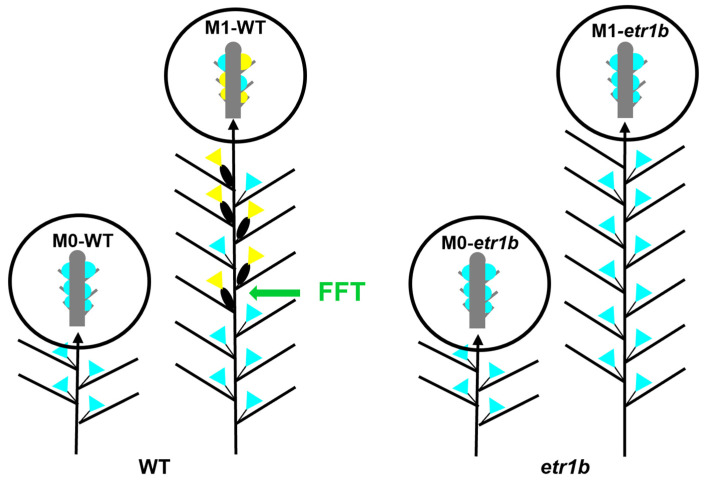
Sex expression of WT and *etr1b* androecious mutant. Schematic representation of the distribution of male and female flowers in WT and *etr1b* mutant plants at M0 stage, 2–4 true leaves, and M1 stage, 10–12 leaves. Blue triangles, male flower; yellow triangles, female flower. The occurrence of the first pistillate flower in the WT is indicated as FFT (Female Flowering Transition). Circles represent a magnification of 1 mm length apical shoots collected to perform RNA-seq analysis. These include the shoot apical meristem (grey), leaf primordium and floral buds with less than 0.5 mm. Blue, male floral buds; yellow, female floral buds.

Two sexual developmental phases can be distinguished in *C. pepo* plants, an initial phase where plants produce a single male flower in each leaf axil, and a second phase in which plants alternate the production of single female or male flowers in the axil of each leaf. The transition between the two phases of plant development, i.e., the time at which the plant starts the production of female flowers, is called the female flowering transition (Figure 1). To gain insight into the molecular mechanisms governing the female flowering transition and female flower specification in *C. pepo*, we took advantage of an ethylene-insensitive androecious mutant (*etr1b*) in which female flowering was completely abolished. The *etr1b* plants develop more than 40 nodes producing only male flowers [20]. The transcriptomic changes occurring in the apical shoots of plants before (M0) and after (M1) the female flowering transition were analyzed with RNA-seq. In M0, both WT and *etr1b* apical meristems were producing male flowers, while in M1 apical meristems, WT plants were producing female and male flowers, but *etr1b* plants were still producing male flowers (Figure 1). This was confirmed by analyzing the apical shoots of both WT and *etr1b* plants under a stereomicroscope. Given that the collected apical shoots had around 1 mm in length and all visible leaves removed, the tissue subjected to RNA-seq was mainly comprising the apical meristem and flower buds at very early stages of development, when the fate of the floral meristem is being determined as a male or as a female flower (Figure 1).

**Figure 2:**
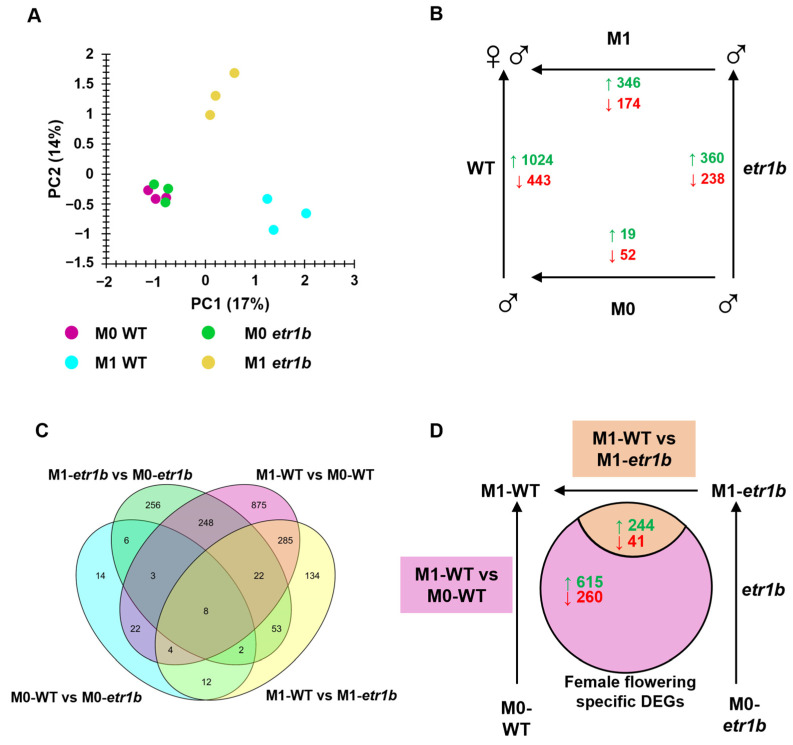
Comparison of transcriptomic profiling between apical shoots of WT and *etr1b* mutant plants at M0 and M1 stages of development. (**A**) Principal Component Analysis of the gene count matrix generated by RNA-seq in three replicates from four apical shoot samples (M0-WT, M1-WT, M0-etr1b and M1-etr1b). The three biological replicates from each genotype and developmental stage show consistent clustering. (**B**) Up- and down-regulated genes in the four different pairwise comparisons. (**C**) Venn Diagrams depicting differentially expressed genes between apical shoots in each comparison analyzed in WT and *etr1b* plants. (**D**) Female flowering-specific DEGs in the apical shoots of WT plants: 875 specific genes from the comparison M0-WT vs. M1-WT, and 285 common between this comparison and M1-WT vs. M1-*etr1b* were selected. Up and down regulated genes in each condition were shown in green and red, respectively.

The 12 cDNA libraries, derived from three replicates of the apical shoots of WT and *etr1b* samples at M0 and M1 stages of development, were sequenced on the NovaSeq 6000 Sequencing System generating paired-end reads of 150 bp (Table 1). After cleaning with FastQC, an average of 12.7 M of clean reads were obtained from each sample, with an average Q30 percentage of at least 94% in all the samples. The average mapped rate against *C. pepo* Genome v4.1 obtained was 97.85%, generating 12.4 M of mapped reads (Table 1), representing 28,221 annotated transcripts in the gene count matrix. The transcript abundance of the RNA-seq data set was used for a Principal Component Analysis (PCA) to test the consistency of the biological replicates (Figure 2A). The PCA plots showed the expected clustering of the three replicates from each genotype and developmental stage of development included in the analysis. The two main components, PC1 and PC2, explained 17% and 14% of gene expression variation. PC1 separated apical shoot samples based on the plant developmental stage (M0 or M1), while PC2 separated the two genotypes at the developmental stage M1 (Figure 2A). At M0, when both genotypes were producing male flowers, samples from WT and *etr1b* were clustered together, indicating low variation in the transcription profiles of the two genotypes. At M1, when WT is already transited to female flowering, the transcriptomic data from WT and *etr1b* were clearly separated (Figure 2A).

### 2.2. Differential Expressed Genes between Genotypes and Developmental Stages

To identify genes that could be potentially involved in *C. pepo* female flowering, we used RNA-seq data set to identify the differential expressed genes (DEGs) in four pairwise comparisons: (i) M0-WT vs. M0-*etr1b*, (ii) M1-*etr1b* vs. M0-*etr1b*, (iii) M1-WT vs. M0-WT, and (iv) M1-WT vs. M1-*etr1b*. More than 2600 DEGs were identified that had a |Log_2_FC| > 1 and an adjusted *p*-value < 0.05 (Figure 2B, Table S1). In the comparison (i) M0-WT vs. M0-*etr1b*, since both WT and *etr1b* plants had the same stage of development and were both producing male flowers, only 19 upregulated and 52 downregulated DEGs were detected (Figure 2B). In the comparison (ii) M1-*etr1b* vs. M0-*etr1b*, the mutant plants were producing male flowers in both, M0 and M1, so the 360 upregulated and 238 downregulated DEGs identified were likely involved in the changes occurring in the mutant plants from M0 to M1 (Figure 2B). None of these DEGs were likely involved in female flowering since this mechanism was suppressed in the mutant. The high number of DEGs detected in the pairwise comparison (iii) M1-WT vs. M0-WT (1024 upregulated and 443 downregulated DEGs) were potentially those regulating the changes in WT plants from M0 to M1 (Figure 2B), including those involved in female flowering transition. Finally, the DEGs of the comparison (iv) M1-WT vs. M1-*etr1b* (346 upregulated and 174 downregulated DEGs) were also likely involved in female flowering transition, but also other changes promoted by the ethylene insensitivity of the mutant *etr1b* (Figure 2B). The ethylene insensitivity of *etr1b*, in fact, not only disrupted female flowering transition, but was also reported to increase plant vigor and salt tolerance [20,28] among other traits that may be also controlled by ethylene. The DEGs affecting these traits may be specific of the fourth pairwise comparison but are not involved in female flowering.

To identify the genes involved in female flowering, Venn diagrams were constructed from the four pairwise comparisons (Figure 2C), selecting those DEGs whose expressions were specifically regulated in the WT from the male to the female phase of development (M1-WT vs. M0-WT) (Figure 2C,D). These included 615 upregulated and 260 downregulated DEGs that are likely involved in female flowering transition and female flower specification and development. In addition, we selected those genes that changed their expression between M1-WT and M1-*etr1b* (244 upregulated and 41 downregulated DEGs) (Figure 2C,D). Given that these genes were deregulated by the ethylene-insensitive mutation *etr1b*, they would represent the genes controlling female flowering with the mediation of ethylene. In total 1160 DEGs, 847 of which annotated in NCBI database, were selected as female flowering-specific DEGs (FFS DEGs) for further analysis.

### 2.3. Ethylene and Other Known Sex-Determining Genes in Cucurbit Species

**Figure 3:**
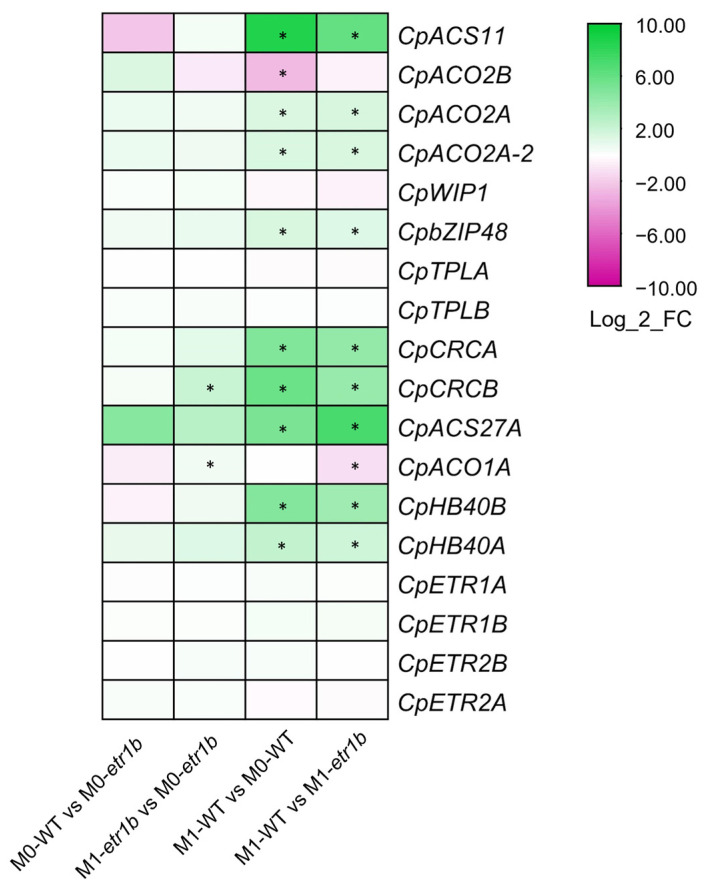
Expression patterns of already reported sex-determining genes in cucurbits. The heatmap represent the average Log_2_FC of each gene in the four pairwise comparisons. M0, apical shoots of plants with four nodes (producing male flowers); M1, apical shoots of plants with 12 nodes (producing female flowers in the WT, but male flowers in the mutant *etr1b*). The scale represents the relative signal intensity of the Log_2_FC values. Asterisks indicates significant differences in gene expression in each comparison (*p* ≤ 0.05).

The heatmap in Figure 3 shows the expression profiles of genes that have been reported to control sex determination in cucurbits, mainly in melon and cucumber. None of the genes changed their expression between WT and *etr1b* at earlier phase of development (M0) when both genotypes are producing male flowers (Figure 3). Only two genes (*CpCRCB* and *CpACO1A*) were significantly upregulated in the mutant apical shoots from M0 to M1 stages (Figure 3). Nevertheless, when the female-producing apical shoots of WT at M1 were compared with male-producing apical shoots of either WT at M0 or *etr1b* at M1, most of the known sex-determining genes were upregulated (Figure 3), indicating that sex determination mechanisms in *C. pepo* are very similar to those described in melon and cucumber. Some of the most important ethylene biosynthesis genes involved in carpel development in the earliest stages of female flower determination are *CpACS11* and *CpACO2A* [9,10]. The two were found to be significantly upregulated in the apical shoots of WT plants upon female flowering, and were therefore detected as female flowering-specific DEGs in both M1-WT vs. M0-WT and M1-WT vs. M1-*etr1b* pairwise comparisons (Figure 3). The next actor in the flower determination pathway, *WIP1*, that works in combination with *bZIP48, CRC,* and *TLP* for the development of male flowers [14,29], did not change its expression profile in the analyzed samples. The *C. pepo TLP* genes, *CpTLPA* and *CpTLPB*, neither displayed expression differences in the analyzed pairwise comparisons. However, the upregulation of the *CRC* and *bZIP48* orthologs, *CpCRCA*, *CpCRCB* and *CpbZIP48*, was notable in WT apical shoots starting to develop female flowers (Figure 3).

Another relevant member of the determination pathway is the ethylene biosynthesis gene responsible for stamen arrest during the development of female flowers, *CpACS27*. This gene was also significantly upregulated in the apical shoots of plants producing female flowers (M1-WT) in comparison to those producing male flowers in the WT (M0-WT) and in the mutant (M1-*etr1b*). *HB40* has been recently reported to be involved in stamen abortion in cucurbits [19]. Interestingly, the orthologs *CpHB40A* and *CpHB40B* also appeared to be upregulated in female producing apical shoots. The ethylene receptor genes *CpETR1A*, *CpETR1B* and *CpETR2B*, which are known to promote carpel development and control the arrest of stamens of female flowers in *C. pepo* [20,21] did not change their expression in the apical shoots producing female flowers, compared with those producing male flowers (Figure 3).

Ethylene-related genes *CpACS27A*, *CpACS11A*, *CpACO2A*, *CpETR1B* and *CpETR1B* were also used for RNA-seq validation. The expression of these genes was compared between WT apical shoots producing female flowers (M1-WT) and those of the WT and the mutants producing only male flowers (M0-WT and M1-*etr1b*). The relative expression profiles obtained by both RNA-seq and qRT-PCR were similar. The linear regression of the data also showed significant positive correlation (R^2^ = 0.8958), confirming the reliability of the RNA-seq data (Figure S1).

### 2.4. Hormonal Pathways Regulating Female Flowering

**Figure 4:**
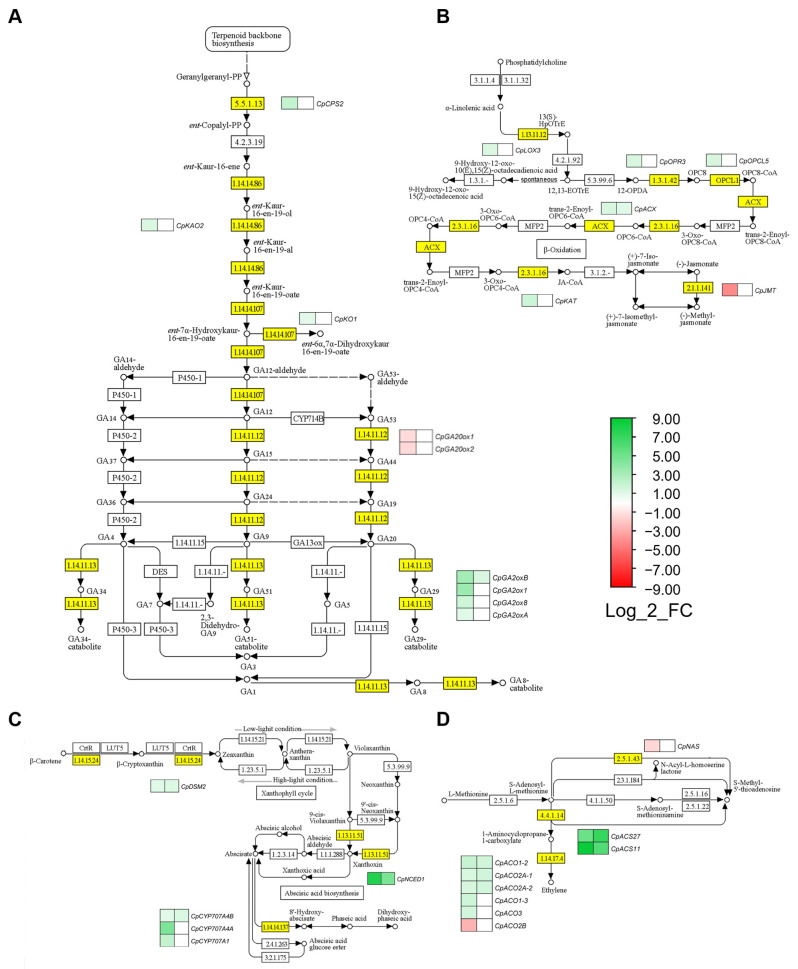
Female flowering-specific (FFS) DEGs in the KEGG maps of different hormone biosynthesis pathways. (**A**) Biosynthesis and activation of gibberellins from diterpenoids. (**B**) Abscisic acid biosynthesis pathway from carotenoids. (**C**) Jasmonate biosynthesis pathway from ⍺-linoleic metabolism and (**D**) thylene biosynthesis pathway from methionine. Yellow box indicates the enzymes encoded by FFS DEGs. Heatmaps next to the yellow boxes represent Log_2_FC of each DEG in two pairwise comparisons: left, specific DEGs in M1-WT vs. M0-WT, common DEGs in M1-WT vs. M0-WT and M1-WT vs. M1-*etr1b* comparisons. The scale represents the relative signal intensity of the Log_2_FC values.

**Figure 5:**
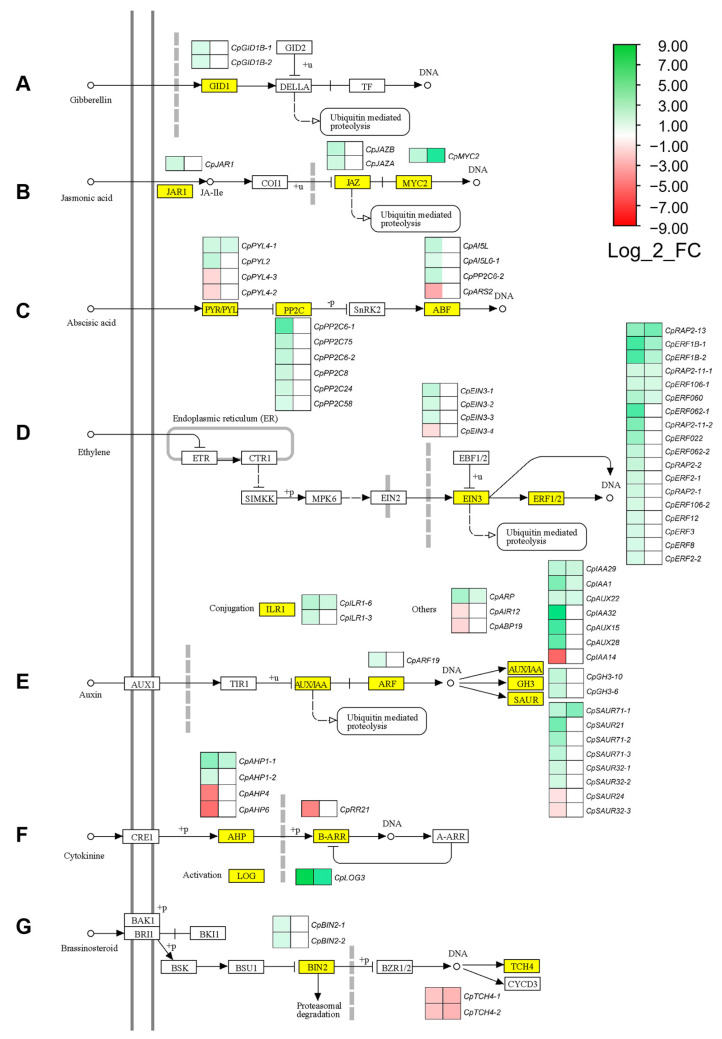
Female flowering-specific (FFS) DEGs in the KEGG maps of different hormone hormones signal transduction pathways. (**A**) Gibberellin, (**B**) Jasmonic acid (Jasmonate), (**C**) Abscisic acid (ABA), (**D**) Ethylene, (**E**) Auxin, (**F**) Cytokinin, and (**G**) Brassinosteroid. Yellow box indicates the enzymes encoded by FFS DEGs. Heatmaps next to the boxes represent Log_2_FC of each DEG in two pairwise comparisons: left, specific DEGs in M1-WT vs. M0-WT, common DEGs in M1-WT vs. M0-WT and M1-WT vs. M1-*etr1b* comparisons. The scale represents the relative signal intensity of the Log_2_FC values.

The 847 selected FFS DEGs were positioned in hormone biosynthesis and signaling pathways of KEGG ontology, determining the possible function of phytohormones in *C. pepo* sex determination and female flower development. FFS DEGS were found in gibberellins (GA), jasmonic acid (JA), abscisic acid (ABA) and ethylene biosynthesis pathways (Figure 4), as well as in the signal transduction pathways of auxins, cytokinins (CK), GA, ABA, ethylene, brassinosteroids (BR) and JA (Figure 5). Interestingly, some of those DEGS comprised those that were regulated by ethylene, changing their expression between M0-WT and M1-WT, but also between M1-WT and M1-*etr1b* apical shoots. The expression of other genes was independent of ethylene, as their expression only changed from M0-WT to M1-WT (Figure 4 and Figure 5).

Female flowering transition and female flower determination was associated with a downregulation of GA, indicating that GA is a negative regulator of female flowering. In fact, genes in earlier steps of the GA biosynthesis (*CpCPS2*, *CpKAO1* and *CpKAO2*) were upregulated (Figure 4A, Table S2). However, in the latter phases of GA biosynthesis (within the activation/deactivation steps), there were four upregulated genes leading to GA deactivation (*CpGA2ox*), and two downregulated genes involved in the production of active GA (*CpGA20ox*) (Figure 4A, Table S2). In the GA signal transduction pathway, female flowering was related to GA receptor genes *CpGID1B-1* and *CpGID1B-2*, which were slightly upregulated during the occurrence of female flowers in WT apical shoots (Figure 5A). However, none of the identified FFS DEGs were deregulated by *etr1b* mutation, with the exception of the GA deactivation gene *CpGA2oxB*, which was slightly upregulated in M1-WT in comparison to M1-*etr1b* (Figure 4A). These data suggests that GA are negative regulators of female flowers determination, independently of ethylene.

Five JA biosynthesis genes (*CpLOX3,* CpOPR3, *CpOPCL5*, *CpACX* and *CpKAT*) were upregulated in the apical shoot upon female flowering. A strong downregulation was found for *CpJMT*, a gene involved in the production of volatile methyl jasmonate and hormone transport (Figure 4B). In the signal transduction pathway, the gene *CpJAR1*, leading the production of JA-Ile active form, and two *CpJAZ* genes, which are transcriptional repressors of JA signaling, were induced (Figure 5B). Only the gene encoding for acyl coenzyme A oxidase (*CpACX*), was additionally induced in the M1-WT compared to M1-*etr1b* (Figure 4B), suggesting that the role of JA in female flowering is independent of ethylene.

A total of 15 ABA genes, five in the biosynthesis pathway and 10 in the perception and signaling pathway, changed their expression upon female flowering. The gene *CpNCED1*, encoding for the ABA primary enzyme 9-cis-epoxycarotenoid dioxygenase, was upregulated in female producing apical shoots (M1-WT) compared to male-producing apical shoots in M0-WT and M1-*etr1b*, indicating that this induction may be mediated by ethylene (Figure 4C). Moreover, four ABA receptor genes (*CpPYLs*), two of which were upregulated and another two downregulated, were found among FFS DEGs. Six *CpPP2C* genes, which are negative regulators of ABA signaling, were also upregulated during female flowering. Finally, there were three upregulated and one downregulated ABF transcription factors in female-producing apical shoots. None of ABA perception and signaling DEGs, with the exception of *CpPYL4-1*, were listed in the ethylene-regulated FFS DEGs (Figure 5C).

Ethylene genes in the list of FFT DEGs validate that ethylene plays a master positive regulatory role in female flower determination. Moreover, the results sustain a feedback regulation of ethylene biosynthesis and perception genes. Thus, there were seven induced genes encoding for most relevant ethylene biosynthesis enzymes ACS and ACO (Figure 4D). As described above, the highly induced *CpACS27* and *CpACS11* genes were already reported as sex-determining genes in cucurbits (Figure 3). Only the ethylene biosynthesis gene *CpACO2B* was repressed during female flowering (Figure 4D). The signal transduction was also mostly activated, with three *CpEIN3s* and 18 *CpERFs* that were induced in female producing apical shoots (Figure 5D). Most of the ethylene signaling genes were found in the list of FFS DEGs that were regulated by ethylene (Figure 5D).

Genes in the signaling pathways of auxins, CK, and BR also changed their expression in the apical shoots during female flowering transition and female flower development. (Figure 5E–G) Two auxin conjugation *CpILR* genes, one *CpARF*, six *CpSAUR*, two *CpGH3* and six *CpAUX/IAA* were upregulated, while one *CpAUX/IAA* and two *CpSAUR* were found to be downregulated in the female producing apical shoot. The gene *CpIAA14* showed a Log_2_FC value of −5.38. Only a few of these auxin signaling genes (*CpAUX22*, *CpIAA9*, *CpIAA29* and *CpSAUR71-1*) were listed within the ethylene-regulated FFS DEGs (Figure 5E), suggesting that auxin may promote female flowering in an ethylene-independent manner. In the CK pathway, four genes of the *AHP* family were either strongly repressed (*CpAHP4* and *CpAHP6*) or moderately induced (*CpAHP1-1*, *CpAHP1-2*), and only *CpAHP1-1* seemed to be regulated by ethylene. The *CpRR21*, which acts as regulator of CK response, was found to be repressed during female flowering. Moreover, there was a strong induction of one Cp*LOG* gene leading to CK activation in the two pairwise comparisons, suggesting that CK activation during female flowering is mediated by ethylene (Figure 5F). Finally, in the BR signaling pathway, two *CpBIN2* genes, which function as negative regulator of BR signaling, were induced from M0 to M1 in WT apical shoots. On the contrary, two members of the *TCH4* family (*CpTCH4-1*, *CpTCH4-2*), encoding for xyloglucan endotransglycosylase, were found to be repressed in the two pairwise comparisons analyzed (Figure 5G), suggesting a repression of BR signaling during female flowering.

### 2.5. Transcription Factors Regulating Female Flowering

Among FFS DEGs, 167 genes were found to encode transcription factors (TF), all of them described in PlantTFDB. Their transcription level changed specifically in the apical shoot of WT plant from M0 to M1 stages of development, but 35% of them (59 TF) were also detected as DEGs in M1-WT vs. M1-*etr1b* comparison and were likely regulated by ethylene. Most of them, with the exception of two, were upregulated upon female flowering (Table 2). TF encoding DEGs belonged to 34 families, with major representation of the families *NAC* (11 DEGs); *ERFs* (seven DEGs); *bHLH* (six DEGs), *bZIP*, *HD-ZIP*, *MYB* (five DEGs) and *C2H2/CH3* (five DEGs) (Table 2). Those TFs that were strongly up or down regulated (Log_2_FC > 4) during female flowering belonged to the TF families bHLH (two DEGs), C2H2 (1), ERF (2), HD-ZIP (2), MIKC_MADS (2), MYB (2)**,** NAC (4) and YABBY (1). Two of these genes, bHLH (111796240) and C2H2 (111789990), showed a Log_2_FC > 6 in female flower producing apical shoots (M1-WT) compared to male flower-producing apical shoots at both M0-WT and M1-*etr1b* (Table 2). Therefore, these two highly induced TF genes are likely regulated by ethylene, and should play an essential function in female flowering. On the other hand, the strongly upregulation and downregulation of other TF genes, including 111780930 (bHLH), 111790146 (C2H2), 111806370 (ERF), 111802196 (G2-like), 111786437 (NAC), 111779181 (ARR-B), 111797571 (MYB) and 111791491 (WRKY), was not mediated by ethylene (Table 2). Some of these TFs were already reported to function as regulators of female flower development in other cucurbits (YABBY 111802629 and DH-ZIP 111781935) [14,19], but the rest represent new transcription factors with a putative regulatory function in *C. pepo* sex determination (Table 2).

### 2.6. Other Transcriptomic Changes Controlling Female Flowering

A total of 483 FFS DEGs were not classified as hormone biosynthesis and signaling genes or as TF coding genes. Of them, 120 DEGs were common in both M1-WT vs. M0-WT and M1-WT vs. M1-MUT pairwise comparison. Many of these genes could be involved in developmental processes associated with female flowering, other than sex determination. However, new candidate genes regulating female flower determination and development might be included in this list. Thus, we investigate the function of those genes that were strongly expressed/repressed (4 ≤ Log_2_FC ≥ 4) in female flower-producing apical shoots in comparison with male-producing apical shoots in the WT (M1-WT vs. M0-WT) or in the mutant plants (M1-WT vs. M1-etr1b). A total of 51 genes were selected (Table S3); 14 of them were upregulated and two downregulated in both pairwise comparisons, whereas 22 were upregulated and 15 downregulated only in the comparison M1-WT vs. M0-WT. The best BLASTP hits against Arabidopsis protein database in both coverage and identity, identified a number of FFS DEGs that were related to stomata aperture, chlorophyll production, amino acid or sugar transport lipid degradation, biosynthesis of alkaloids, carotenoids, flavonoids, wax or lignin. Additionally, valuable candidate genes were found that may be associated with the differential development of female and male flowers (Table 3, Table S3). Among them we found five upregulated genes: one is a bidirectional sugar transporter SWEET4-like, one annotated as *protein FANTASTIC FOUR 2-like*, two *receptor-like protein kinase FERONIA,* one *glu S.griseus protease inhibitor-like isoform X1* and two downregulated genes, *AT-hook motif nuclear-localized protein 22-like* and *protein RSI-1-like* (Table 3). Notably, some of them respond to ethylene sensitivity.

Taken together, our results confirm that previously discovered sex-determining genes in melon and cucumber may have the same function in *C. pepo*. At the same time, we propose a number of interesting candidates in the control of the female flowering transition and the differential development of male and female flowers in *C. pepo*. A model is included integrating the function of ethylene and other hormonal cues, as well as new relevant TF genes in the onset and early development of the female flower of *C. pepo*.

## 3. Discussion

In the monoecious plant *C. pepo*, the transition between the male and female phases of sexual development (female flowering transition) is a crucial determinant of reproductive success and crop production. The goal of this study was to uncover the molecular mechanisms that underlie female flowering transition and sex determination, controlling the specification and development of the female flowers. To do this, we have taken advantage of the ethylene-insensitive mutant *etr1b*, which has completely suppressed female flowering transition as well as the determination mechanisms leading to female flower development [20].

### 3.1. Ethylene Genes Regulating Female Flowering in C. pepo

RNA-seq approach allowed the identification of the specific transcriptomic changes occurring in the plant apical shoot from the male to the female flower of development in WT plants. The transcriptomic changes occurring in the apical shoots of the androecious mutant *etr1b* during the same phenological stages allowed to filter out those DEGs that were involved in developmental and physiological processes other than female flowering. After that we selected 1160 female flowering specific DEGs. In addition, given that the androecious phenotype of *etr1b* is caused by a deficiency in ethylene sensitivity [20], the transcriptomic changes between WT apical shoot at female flowering and those of the mutant at the same phenological stage, permitted the identification of 285 female flowering specific DEGs whose expression is likely mediated by ethylene. In accordance with previous transcriptomic studies in other cucurbit species [48,49,50], the performed transcriptomic study has indicated that ethylene-related genes play a significant role in female flowering, but there are other hormones and molecular mechanisms occurring in the apical shoot during the specification and early development of female flowers.

Ethylene is a key hormone in the control of sex determination in cucurbits [1]. Our transcriptomic analysis confirmed that there are a number of genes in the ethylene biosynthesis and signaling pathway that were upregulated in the apical shoot of *C. pepo* plants upon female flowering. The induction of *CpACS11* and *CpACO2* genes, whose orthologs in melon and cucumber functions as promoters of carpel development during the specification of the female floral meristem [9,10], clearly indicate that these genes have also a positive role during female flower determination in *C. pepo*. Given that this induction appears to be mediated by ethylene, data also showed a feedback regulation of ethylene biosynthesis genes involved in carpel development, as has been previously proposed [6]. As expected, other late acting ethylene biosynthesis genes like *CpACS27*, involved in stamen arrest during the development of female flowers [7], were also highly upregulated in the apical shoot upon female flowering. These results validate the effectiveness of our analysis to detect those genes that are actually involved in the differential development of female and male flowers in *C. pepo*. *CpWIP1*, the key transcription factor responsible for the arrest of carpel development in the male flowers of melon and cucumber [12] was not differentially expressed in the apical shoot of *C. pepo* during female flowering. However, the orthologs of *CRC*, a gene that is necessary for carpel development in melon and cucumber [14], dramatically increase its expression in female apical shoots of *C. pepo*. This data suggests that the activation of *CpCRCA and CpCRCB* is also essential in *C. pepo* for the determination of the floral meristem into a female flower. The same is true for the recently described transcription factor coding gene *CmHB40* of melon [19], which was found to be also regulated by ethylene in the apical shoot of *C. pepo* and is required for the proper arrest of stamens in female development.

### 3.2. Other Hormone-Related Genes Regulating Female Flowering in C. pepo

**Figure 6:**
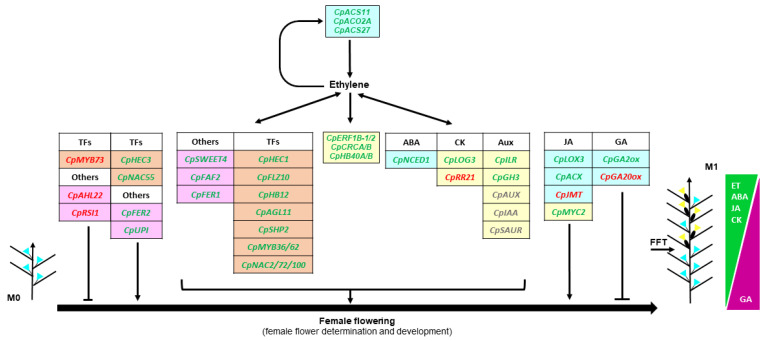
Model integrating hormonal cues, transcription factors and other genes in the control of female flowering in *Cucurbita pepo*. Tables contain genes found regulated under the pairwise comparisons: M1-WT vs. M0-WT and M1-WT vs. M1-*etr1b*. The induced/repressed genes in the second comparison depend on ethylene. Background colors indicate hormone biosynthesis pathway (blue), hormone signaling and response (yellow), transcription factors (TF) not included in hormonal regulation (orange) and other novel female flowering related genes (pink). Upregulated genes are highlighted in green, downregulated genes in red, gene families with both up- and downregulated genes are indicated in grey. Arrows indicate positive regulation/interaction and blocking-lines negative regulation.

On the base of hormone-related FFS DEGs, we have proposed a model for the hormonal regulation of female flowering in *C. pepo* (Figure 6). Transcriptomic changes between male and female producing apical shoots indicate that, in addition to ethylene, the hormones auxin, CK, BR, JA and ABA have a positive role on female flowering in *C. pepo*. This process includes the specification of the female floral meristem and/or the early stages of female flower development. No FFS DEG was found to be related to auxin biosynthesis, but several auxin-induced genes were found to be upregulated in the apical shoots of the WT plants during female flowering, suggesting that femaleness is associated with a high auxin level in the shoot apex. This was the case for the genes *CpILR* and *CpGH3*, which are involved in auxin conjugation and the maintenance of auxin homeostasis [51]. The same pattern was observed for several auxin signaling genes from the multigenic families *CpIAA*/*AUX* and *CpSAUR*. Previous studies have described the relationship between auxins and ethylene. Indoleacetic Acid (IAA) treatments induce the expression of *ACS* genes and the production of ethylene in cucumber [25]. Moreover, our and other transcriptomic studies in tomato and cucumber [52,53] indicated that auxin signaling genes, specially *IAA29*, are regulated by ethylene. Therefore, it is likely that the action of auxin on female flowering of *C. pepo* depends on the crosstalk between these two hormones. The minor effect of BR on cucumber and squash sex expression was reported to be also mediated by ethylene [24,54]. In our RNA-seq analysis, however, we found that two *CpBIN2* genes, which are negative regulators of BR signaling, were upregulated, while two auxin- and BR-induced *CpTCH4* genes [55], were downregulated in the shoot apex during female flower production. This result does not clarify the function of BR in the determination and development of the *C. pepo* female flower.

Most of the genes encoding for enzymes in the JA biosynthesis and signaling pathways were upregulated in female-producing apical shoots, and their transcription changes appears to be independent of ethylene (Figure 6). Only *CpJMT*, involved in hormone transport by generating the volatile derivative Methyl-Jasmonate (MeJa) [56], showed a negative Log_2_FC in female producing apical shoots. During later stages of corolla development, the female flower of *C. pepo* requires more JA than the male flower to reach maturation and to open [57], but up to now, no study had reported the role of JA in early events modulating the differential development of male and female flowers of cucurbits.

The role of ABA in the differential development of female and male flowers of cucurbits is not clear. The cold-induced femaleness of cucumber plants seems to be mediated by both sugar and ABA signaling pathways [58,59]. ABA signaling genes *CsABI1* and *CsABI2* (protein phosphatase 2C gene family) were also found to be differentially expressed in female and male flowers of cucumber [60]. Moreover, it is likely that ABA exerts its action through the regulation of *CTR1* gene in the ethylene signaling pathway [61]. Our RNA-seq analysis also indicates ethylene-mediated high upregulation of ABA important biosynthetic gene *CpNCED1* in female flowering producing apical shoots, suggesting a collaborative positive role of ethylene and ABA during female flower initiation and development (Figure 6).

Finally, CK has a feminizing effect in other monoecious species [62,63,64], but their role in *C. pepo* sex determination was unknown. Our results suggest that CK also acts as a positive regulator of female flowering, since *CpLOG3*, involved in the production of the active forms of CKs [65], is highly upregulated during female flowering in response to ethylene (Figure 6). However, the regulation of CK signal transduction pathway is not so clear, as *CpAHP* genes show both upregulation and downregulation, and the transcriptional regulator *CpRR21* is highly repressed. The best hit in Arabidopsis, ARR2, participates in signal transmission of CK and ethylene and plays roles in numerous developmental processes [66,67]. Interestingly, loss-of-function *arr2* mutant displayed early flowering [66]. On the other hand, GAs have a negative effect on female flowering, but promote the development of male flowers. In fact, GA promotes maleness in different cucurbit species [26,68,69]. The downregulation of GA biosynthesis gene *CpGA20ox* and the upregulation of deactivation gene *CpGA2ox*, indicate that the content of active GA active is reduced in shoot apex upon female flowering.

### 3.3. Transcription Factors Associated with Female Flowers Production

Several transcription factors have been identified to change their expression upon female flowering in *C. pepo*. Most of these female flower-specific (FFS) DEGs were found to be regulated by ethylene and their expression level in the apical shoots of the *etr1b* androecious plants was opposite to that found in WT plants upon female flowering. Some of the upregulated NAC-type TFs are associated with ABA response (*NAC55*, [47]), and others like *NAC72* (111800590, 111787610), *NAC2* (111808051), and *NAC100* (111791093) were related to the ethylene response [43,46]. Two MYB were strongly upregulated, the first is *MYB36* gene (111788261) with unknown functions, while the second, *MYB62* (111809067), appears to affect flowering in Arabidopsis by regulating GA under phosphate starvation conditions [39,40]. On the other hand, *MYB73*, involved in salt stress and auxin response [41,42] was highly repressed during female flowering. Two homeotic genes, *AGL11* (111789058) and *SHP2* (111782688) were also upregulated in squash female flowering. AGL11 is involved in the last stages of ovule and seeds development in Arabidopsis, tomato or grape [35,36,37], whereas SHP2 regulates carpel development and is involved in CK feedback regulation by A-ARR and B-ARR TFs in Arabidopsis [38]. Among the TF genes that greatly change their expression during female flowering, it is worth mentioning a bHLH TF (111796240), annotated as *HEC1*. This gene has been described as a regulator of gynoecium development in Arabidopsis [31]. Another interesting FFT DEG is annotated as a C2H2 *zinc finger protein 10-like*. This protein regulates plant development and plays a negative role during ABA signaling by repressing *SnRK1*. It is expressed in the pistils and anthers of the flower bud of Arabidopsis. Its possible role in the differential development of male and female flowers in *C. pepo* has not been reported previously [33,34].

### 3.4. Other Actors Regulating Female Flowering Transition Process

Outside of the described categories, other five DEGs have been found to have an effective role on the specification and development of female flowers in *C. pepo*. Three of them were found to be regulated by ethylene in our study. The first is a bidirectional sugar transporter SWEET4-like, that generally controls sugar metabolism during flowering or fruit development [70], and seed filling in maize and rice [71]. The second is the protein FANTASTIC FOUR 2-like, whose expression increased during the flowering transition in Arabidopsis [72]. The third one is the receptor-like protein kinase FERONIA, which is a positive regulator of flowering [73] and is involved in JA signaling repression [73,74]. Two other genes were not found to regulate ethylene in our study. One also encodes for a receptor-like protein kinase FERONIA family and the second encodes for a *glu S.griseus* protease inhibitor-like isoform X1, which best hit of Arabidopsis encodes for UNUSUAL SERINE PROTEASE INHIBITOR. A mutant of the latter presents delayed flowering and changes in the expression of *SOC1* and *MAF1* floral transition regulators [75]. Finally, the downregulated genes for AT-hook motif nuclear-localized protein 22-like and protein RSI-1-like are also related to flowering transition in Arabidopsis. AT-hook motif nuclear-localized protein 22-like (AT3G60870) participates in flowering initiation by modifying *FLOWERING LOCUS T* chromatin [76]. *RSI-1-like* (AT3G09950) is allelic to *FLOWERING LOCUS D* that in turn represses *FLOWERING LOCUS C*, that binds and represses *SOC1* and *FT* [77,78]. None of these genes were reported to have a role in cucurbit sex determination, but they are good candidates for forthcoming functional studies.

Our results suggest that the control of the female flowering production in *C. pepo* is consistent with what occurs in other cucurbits, but many novel factors have been identified in this work that play a crucial role in the earlier mechanisms of cucurbits sex determination. The identification and future characterization of these genes may shed light on the complex regulatory network that defines the fate of these meristems to become a male or female flower during the development of the plant.

## 4. Materials and Methods

### 4.1. Plant Material

The ethylene-insensitive mutation *etr1b* affects the ethylene receptor gene *CpETR1B* and confers androecy [20]. Mutant and WT plants were used to study the molecular mechanisms that control sex determination in *C. pepo*. Given the female sterility of the mutant, WT and *etr1b* homozygous plants were selected from a BC3S1 population derived from the selfed offsprings of heterozygous BC3 plants. The genotype of the segregating population was assessed with Taq-man probes. The multiplex PCR reactions were done with the SensiFAST™ Probe No-ROX Kit (Bioline, London, UK), a set of forward and reverse primers amplifying the polymorphic sequence, and two allele-specific probes. The PCR conditions were established in base to primers annealing temperature following the requirement of the fabricant. The experiment was performed in the Rotor Gene Q platform (Qiagen, Hilden, Germany) thermocycler. Genotyping primers are shown in Table S3.

WT and *etr1b* plants were grown in a chamber under controlled conditions: 25 °C of temperature and photoperiod of 16/8 h day/night in spring of 2019. The plant apical shoots were collected at two stages of plant development: (i) M0, plants with 2–4 leaves, where the apical shoots of both WT and *etr1b* were producing only male flowers, and (ii) M1, plants with 10–12 leaves, where the WT apical shoot was producing female and male flowers, but that of *etr1b* was producing male flowers. The apical shoot of each plant was then dissected under a stereomicroscope, removing all visible leaves and flowers, and leaving only the apical meristem, the smallest leaf primordia, and the smallest floral buds. The tissue was quickly frozen in liquid N2 and stored at −80 °C until use.

### 4.2. RNA Isolation and Sequencing

The frozen apical shoots were ground using stainless steel beads, previously cooled with dry ice. Three biological replicates were included in each studied condition. RNA of each replicate was extracted by using the GeneJET Plant RNA Purification Kit (Thermo Fisher Scientific^®^, Waltham, MA, USA) following the manufacturer’s protocol. Complementary DNA was then synthesized using a RevertAid RT Reverse Transcription Kit (Thermo Fisher Scientific^®^, Waltham, MA, USA). After all the purification steps, RNA was eluted in nuclease-free water and immediately prepared for sequencing.

Library construction and sequencing were performed by Novogene, Beijing, China. The sequencing platform used was the NovaSeq 6000 Sequencing System (Illumina, San Diego, CA, USA), generating 150 pb pair-end reads and obtaining 6 Gb of raw data per sample.

### 4.3. Bioinformatic Analysis and Graphic Representations

The quality of raw data was first checked with FASTQC [79] and then cleaned of adapters, contaminants, and low-quality reads with TRIMMOMATIC [80] and FASTQ-SCREEN [81] programs. After a second FASTQC analysis, reads were aligned against the reference genome of *Cucurbita pepo* by HISAT2 [82], using STRINGTIE [83,84] to obtain the count of transcripts. The quantification of these samples was performed by FEATURECOUNTS. R packages EDGER [85,86] and LIMMA-VOOM [87,88] were used to normalize data and obtain the Differentially Expressed Genes (DEGs) between samples using the included function TOPTABLE. Adjusted *p*-value for each gene was calculated using the Benjamini and Hochberg (BH) method [89]. Genes with a value of |Log_2_FC| > 1 and an associated adjusted *p*-value < 0.05 were selected as Differentially Expressed Genes (DEGs). The selected DEGs were then graphically compared using Venn diagrams and represented by a heat map constructed with TBtools [90].

### 4.4. RNA-Seq Validation by Quantitative RT-PCR

Five ethylene-related genes were selected for RNA-seq data validation by qRT-PCR on three biological replicates of the conditions included for RNA-seq. Total RNA was isolated with the GeneJET Plant RNA Purification Kit (Thermo Fisher Scientific^®^, Waltham, MA, USA). Then, cDNA was synthetized with the cDNA RevertAidTM kit (Thermo Fisher Scientific^®^, Waltham, MA, USA). qRT-PCR was carried out in a 10 μL total volume with 1× Top Green qPCR Super Mix (Bio-Rad^®^, Hercules, CA, USA) on the thermocycler of the CFX-96 Touch Real-Time PCR Detection System (Bio-Rad^®^, Hercules, CA, USA). Gene expression values were calculated using the 2^–ΔΔCT^ method [91]. Two constitutive genes were used as internal reference, *CpEF1α* and *CpACT*. Primers for gene expression analysis are shown in Table S3.

### 4.5. Functional Analysis

NCBI database (https://www.ncbi.nlm.nih.gov/, accessed on 20 July 2023) database was used for gene annotation. Gene function annotation and pathway analysis were carried out using the Kyoto Encyclopedia of Genes and Genomes [92] and represented by KEGG mapped color (https://www.genome.jp/kegg/mapper/color, accessed on 25 September 2023). Transcription factors were identified and classified in the Plant Transcription Factor Database v5.0 (http://planttfdb.gao-lab.org/, accessed on 2 October 2023).

## 5. Conclusions

Our study sheds light on the intricate molecular mechanisms governing the transition from male to female flowering in *C. pepo*. The ethylene-insensitive mutant *etr1b* served as a valuable tool to identify female flowering-specific differentially expressed genes (DEGs). The transcriptomic analysis revealed the prominent role of ethylene in sex determination, as evidenced by the upregulation of key genes in the ethylene biosynthesis and signaling pathways during female flowering. Additionally, the study indicates that female flower specification and development depends on a more complex hormonal regulatory network, which involves auxin, cytokinin, jasmonic acid, gibberellins and ABA in an ethylene-dependent or independent manner. Notably, transcription factors and other genes with diverse functions were identified as potential regulators of sex determination in *C. pepo*, emerging as promising candidates for further in-depth studies.

## Figures and Tables

**Table 1:** RNA sequencing data.

Genotype	Develop. Stage	No Sample	Clean Reads	Q20 (%)	Q30 (%)	GC (%)	Mapping (%)	Mapped Reads
WT	M0	1	12,863,488	97.81	93.73	44.92	98.13	12,622,941
		2	13,356,496	97.87	93.88	45.02	97.90	13,076,010
		3	12,735,924	97.98	94.36	45	97.41	12,406,064
	M1	1	11,555,691	97.9	94.08	44.91	97.54	11,271,421
		2	16,244,907	97.91	93.91	45.23	97.68	15,868,025
		3	11,244,769	98	94.29	45.16	98.31	11,054,732
*etr1b*	M0	1	12,843,946	97.66	93.43	44.91	97.42	12,512,572
		2	12,246,854	97.95	94.2	44.85	98.34	12,043,556
		3	14,258,714	98	94.22	45.16	98.14	13,993,502
	M1	1	11,358,393	97.97	94.21	45.01	97.96	11,126,682
		2	12,456,499	97.89	94.03	44.73	97.57	12,153,806
		3	11,225,221	97.92	94.1	44.85	97.77	10,974,899

RNA-seq was performed in apical shoot samples from WT and *etr1b* mutant plants at M0 and M1 stages of development. M0, apical shoots of plants with 2–4 leaves, before female flowering. M1, apical shoots of plants after female flowering, WT plants are producing both male and female flowers, but male flowers are still in the male phase of development. Q20 (%) indicates an error rate of 1 in 100 (meaning every 100 bp sequencing read may contain an error), with a corresponding call accuracy of 99% and Q30 (%) indicates an error rate of 1 in 1000 with a corresponding call accuracy of 99%, respectively. GC (%) indicates the percentage of G and C in the total bases.

**Table 2:** List of selected DEGs that encode different transcription factors in the comparisons M1-WT vs. M0-WT and M1-WT vs. M1-*etr1b*.

TF Family	Gene ID	Annotation	M1-WT vs. M0-WT	M1-WT vs. M1-*etr1b*	Putative Function	Organism	**Name**
ARR-B	111779181	two-component response regulator ORR21-like	−4.36	0.00	Cytokinin signaling [30]	*A. thaliana*	*CpORR21*
bHLH	111796240	transcription factor HEC1-like	8.27	7.08	Control of gynoecium development via auxin and cytokinin response [31]	*A. thaliana*	*CpHEC1*
bHLH	111800158	transcription factor MYC2-like	1.73	4.32	Jasmonate signaling [32]	*A. thaliana*	*CpMYC2*
bHLH	111780930	transcription factor HEC3-like isoform X3	5.46	0.00	Control of gynoecium development via auxin and cytokinin response [31]		*CpHEC3*
C2H2	111789990	zinc finger protein 10-like	7.39	5.44	Plant growth and development [33], ABA signaling [34]	*A. thaliana*	*CpFLZ10*
C2H2	111790146	zinc finger protein 11-like	4.34	0.00			*CpFLZ11*
ERF	111791372	ethylene-responsive transcription factor 1B	4.45	2.64			*CpERF1B-1*
ERF	111800217	ethylene-responsive transcription factor 1B-like	4.36	2.15			*CpERF1B-2*
ERF	111806370	ethylene-responsive transcription factor ERF062-like	4.34	0.00			*CpERF62*
G2-like	111802196	transcription factor HHO2-like	5.08	0.00			*CpHHO2*
HD-ZIP	111781935	homeobox-leucine zipper protein ATHB-40-like	4.82	3.76	Stamen abortion in bisexual flowers [19]	*C. melo*	*CpHB40A*
HD-ZIP	111801688	homeobox-leucine zipper protein ATHB-12-like	6.72	2.98			*CpHB40B*
MIKC_MADS	111789058	agamous-like MADS-box protein AGL11 isoform X2	5.94	4.04	Seed development [35,36,37]	*A. thaliana*, *S. lycopersicum*, *V. vinifera*	*CpAGL11*
MIKC_MADS	111782688	floral homeotic protein AGAMOUS-like	5.57	3.66	Cytokinin regulation [38]	*A. thaliana*	*CpSHP2*
MYB	111788261	transcription factor MYB36-like	5.98	3.25			*CpMYB36*
MYB	111809067	transcription factor MYB62-like	6.62	2.99	Flower development via gibberellin biosynthesis and activation under phosphate starvation conditions [39] and gibberellin signaling [40]	*A. thaliana*	*CpMYB62*
MYB	111797571	transcription factor MYB73-like	−4.22	0.00	Auxin response [41,42]		*CpMYB73*
NAC	111800590	NAC domain-containing protein 72-like	5.56	4.55	Transcription factor RD26. Abiotic stress via ABA response [43]	*A. thaliana*	*CpNAC72-1*
NAC	111808051	NAC domain-containing protein 2-like	4.02	3.81	Transcription factor ATAF1. ABA homeostasis [44,45]	*A. thaliana*	*CpNAC2*
NAC	111787610	NAC domain-containing protein 72-like	4.28	3.58	Transcription factor RD26. Abiotic stress via ABA response [43]	*A. thaliana*	*CpNAC72-2*
NAC	111791093	NAC domain-containing protein 100-like	5.23	3.05	Leaf senescence via module EIN2-EIN3-miR164-NAC2 [46]	*A. thaliana*	*CpNAC100*
NAC	111786437	NAC domain-containing protein 55-like	4.47	0.00	Stress response mediated by ABA [47]	*A. thaliana*	*CpNAC55*
WRKY	111791491	probable WRKY transcription factor 48	−4.67	0.00			*CpWRKY48*
YABBY	111802629	protein CRABS CLAW	4.91	4.19	Carpel development [14]	*C. melo*	*CpCRCA*

Up-regulated DEGs are highlighted in green and down-regulated DEGs in red. Higher to lower intensities of color correspond to higher to lower values on Log_2_FC, respectively.

**Table 3:** List of candidate genes linked to female flowering.

		Log_2_FC		1st Hit in *A. thaliana*			
Gene ID	Annotation	T1-WT vs. T0-WT	T1_WT vs. T1_*etr1b*	Coverage (%)	Homology (%)	TAIR Gene ID	**Name**
111796025	bidirectional sugar transporter SWEET4-like	3.63	4.12	88	55.13	AT3G28007	*CpSWEET4*
111811228	protein FANTASTIC FOUR 2-like	4.23	1.94	69	40.35	AT4G02810	*CpFAF2*
111790342	receptor-like protein kinase FERONIA	4.80	1.72	96	48.20	AT3G51550	*CpFER-1*
111790341	receptor-like protein kinase FERONIA	4.27	0.00	96	48.20	AT3G51550	*CpFER-2*
111806461	*glu S.griseus* protease inhibitor-like isoform X1	4.00	0.00	100	50.00	AT5G43580	*CpUPI*
111779138	AT-hook motif nuclear-localized protein 22-like	−4.97	0.00	100	52.38	AT3G60870	*CpAHL22*
111809214	protein RSI-1-like	−6.24	0.00	72	63.89	AT3G09950	*CpRSI1*

Up-regulated DEGs are highlighted in green and down-regulated DEGs in red. Higher to lower intensities of color correspond to higher to lower values on Log_2_FC, respectively.

## Data Availability

All relevant data can be found within the manuscript and its supporting materials. All of the raw reads generated in this study have been deposited in the public database of National Center of Biotechnology under BioProject PRJNA1019138.

## Supplementary Materials

The supporting information can be downloaded at: https://www.mdpi.com/article/10.3390/ijms242417195/s1.
