# Supplementary material for: Comparative RNA-Seq Analysis between Monoecious and Androecious Plants Reveals Regulatory Mechanisms Controlling Female Flowering in Cucurbita pepo
Source: Int J Mol Sci. 2023 Dec 6;24(24):17195. doi: 10.3390/ijms242417195 (PMC10743737; doi:10.3390/ijms242417195)
Supplement: Supplementary file 1 [file ijms-24-17195-s001.zip › Supplementary Figures.pptx]

## Slide 1
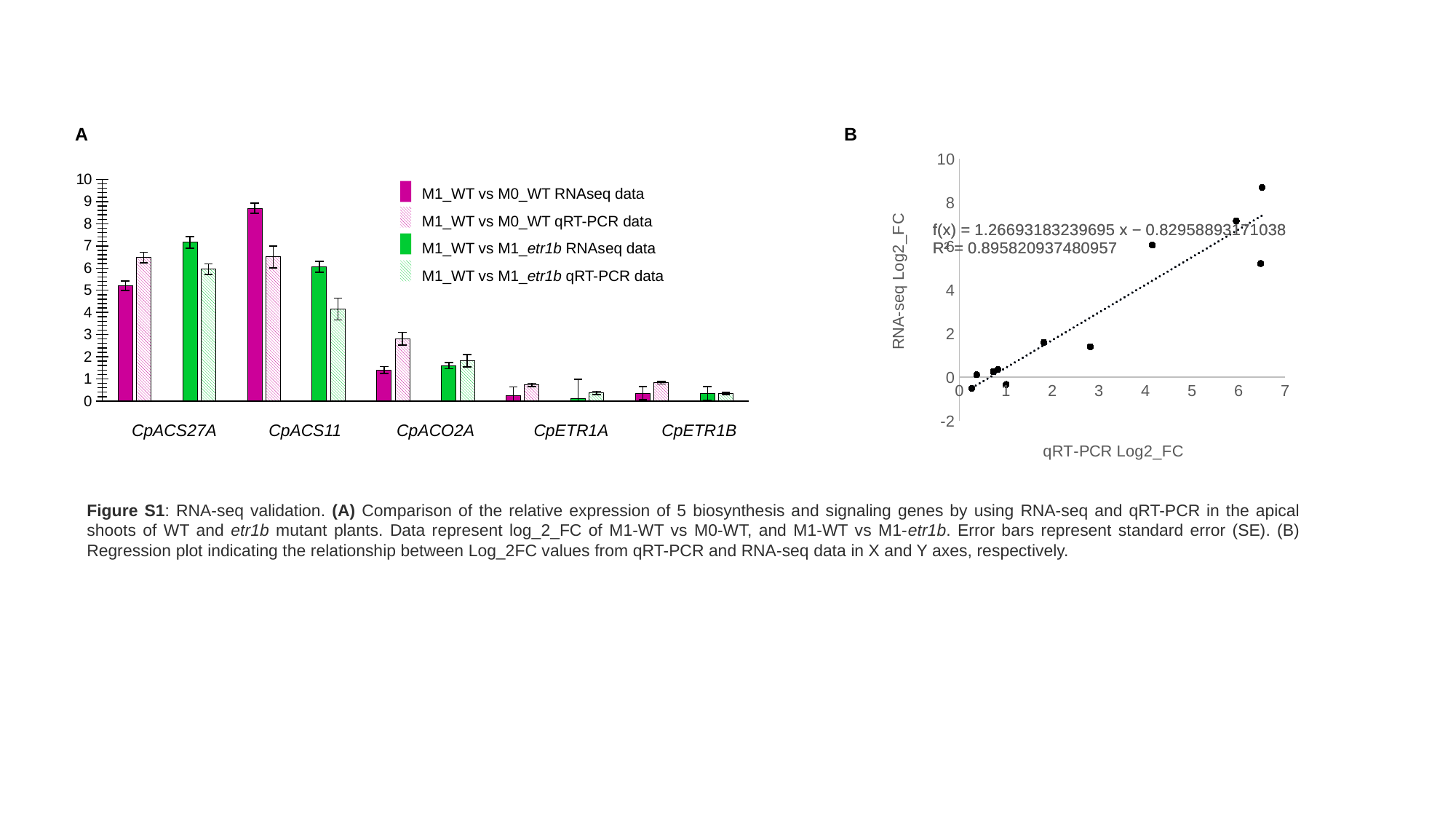

A
B
### Chart
| Category | |
|---|---|
### Chart
| Category | RNA-seq | (qRT)-PCR |
|---|---|---|
| CpACS27 | 5.21000071400591 | 6.473333333333332 |
| | 7.16215531948392 | 5.946666666666666 |
| CpACS11 | 8.69903917087719 | 6.503333333333331 |
| | 6.05769624942498 | 4.146666666666663 |
| CpACO2 | 1.40156980408122 | 2.8133333333333317 |
| | 1.59801339665913 | 1.8133333333333308 |
| CpETR1A | 0.257068227077666 | 0.7299999999999996 |
| | 0.113360716450782 | 0.36999999999999933 |
| CpETR1B | 0.35435252668612 | 0.826666666666667 |M1_WT vs M0_WT RNAseq data
M1_WT vs M0_WT qRT-PCR data
M1_WT vs M1_etr1b RNAseq data
M1_WT vs M1_etr1b qRT-PCR data
CpACS27A
CpACS11
CpACO2A
CpETR1A
CpETR1B
Figure S1: RNA-seq validation. (A) Comparison of the relative expression of 5 biosynthesis and signaling genes by using RNA-seq and qRT-PCR in the apical shoots of WT and etr1b mutant plants. Data represent log_2_FC of M1-WT vs M0-WT, and M1-WT vs M1-etr1b. Error bars represent standard error (SE). (B) Regression plot indicating the relationship between Log_2FC values from qRT-PCR and RNA-seq data in X and Y axes, respectively.
